# Evaluation of circulating extracellular vesicles and miRNA in neutered and obese female dogs

**DOI:** 10.1038/s41598-022-20523-x

**Published:** 2022-09-30

**Authors:** Paola Caroline da Silva Nunes, Rosane Mazzarella, Juliano Coelho da Silveira, Deise Carla Almeida Leite Dellova

**Affiliations:** Department of Veterinary Medicine, Faculty of Animal Science and Food Engineering, University of São Paulo, Pirassununga, 13635-900 Brazil

**Keywords:** Molecular biology, Physiology, Endocrinology

## Abstract

Adipose tissue is a metabolic and endocrine organ, and its adipocytes can synthesize and secrete extracellular vesicles (EVs), thus allowing intercellular communication. EVs are nanoparticles that transport lipids, proteins, metabolites, and nucleic acids (mRNA and microRNAs). MicroRNAs (miRNAs) are small, non-coding RNAs that regulate gene expression. miR-132, miR-26b, and miR-155 are associated with obesity, lipid metabolism and adipogenesis. The aim of this study was to evaluate the enriched EVs fraction containing miRNAs (miR-132, miR-26b, and miR-155) in serum from obese female dogs. Thirty-two neutered females in good general condition were recruited, including 21 obese and 11 healthy controls. The initial evaluation of the females included a general physical examination and laboratory tests. Small EVs (sEVs) were isolated from whole blood by serial centrifugation and ultracentrifugation, and nanoparticle analysis was used to determine the size and concentration of serum sEVs. miRNAs were extracted from sEVs enriched fraction and analyzed by real-time polymerase chain reaction. Obese female dogs with hypertriglyceridemia showed an increase in the sEVs concentration and in the expression of miR-132 and miR-26b in sEVs enriched fraction. No changes were observed in the group of obese female dogs with normal serum biochemical profile and in relation to miR-155 expression. These results suggest that obese female dogs with hypertriglyceridemia may present alterations in sEVs and in the expression of miRNAs related to lipid metabolism and adipogenesis.

## Introduction

Adipose tissue is a metabolic and endocrine organ that actively participates in energy regulation, carbohydrate and lipid metabolism, inflammation, and coagulation cascades. This tissue has the function of storing and releasing energy, participates in the immune response, and performs cell-to-cell communication through the release of hormones, cytokines, and growth factors^1, 2^. Furthermore, adipose tissue can synthesize, release, and transfer extracellular vesicles (EVs) to cells that could be regulated by it (target cells)^3^.

EVs are nanoparticles synthesized by different cells, and secreted into the extracellular environment, thus communicating with different organs. They can be classified according to their biogenesis as follows: apoptotic bodies (> 1000 nm), microvesicles (100–1000 nm), and exosomes (50–150 nm)^4^. Due to the difficulty in establishing their exact origin, the International Society of Extracellular Vesicles also classifies EVs according to size: small EVs (sEVs) (< 200 nm) and large EVs (> 200 nm)^5^.

Intercellular communication, genetic exchange, and antigen presentation occur through the content of EVs, including proteins, lipids, and nucleic acids, such as mRNA and microRNAs (miRNAs)^6^. The presence of EVs in different body fluids and their easy enrichment allows the analysis and characterization of their content and associated molecules, being defined as less invasive biomarkers, and their extraction as “liquid biopsy”^7^.

miRNAs are a highly conserved group of small non-coding RNAs approximately 22 nucleotides in length that regulate gene expression. miRNAs can repress translation and/or initiate target mRNA degradation through base pairing at complementary sites within mRNA, thus controlling diverse biological processes in different tissues^8, 9^.

Obesity is defined as excess body fat resulting from a positive energy balance when there is high consumption but low energy expenditure, which may also result in one or more comorbidities. Currently, obesity is treated as a complex clinical syndrome with a multifactorial etiology, with emphasis on positive energy balance, genetic, metabolic, and endocrine factors, as well as the use of drugs that induce excessive food intake, such as the corticosteroids^10^. A study conducted in a big Brazilian city showed a prevalence of 40.5% in overweight and obese dogs, with a higher prevalence in spayed female dogs^11^.

Adipocytes have the ability to release EVs and studies carried out in humans and mice have shown that obesity can alter the size and number of adipocyte-derived EVs, as well as the expression of molecules associated with these EVs, such as miRNAs^12–14^. In this sense miR-132, miR-26b, and miR-155 are associated with obesity, lipid metabolism, adipogenesis, macrophage infiltration, inflammation, and insulin secretion, among others^15–19^.

There is no evidence from studies in female dogs relating obesity to the analysis of serum EVs and the profile of miRNAs in enriched EVs fraction. Therefore, the objective of this study was to obtain a sEVs enriched fraction from serum samples of female dogs with a normal body condition score (BCS) and obesity, using differential centrifugation followed by ultracentrifugation technique, to later characterize these nanoparticles (size and concentration) and evaluate the expression of obesity associated miR-132, miR-26b, and miR-155 in the sEVs enriched fraction, using quantitative real-time polymerase chain reaction (PCR).

## Results

### Obese female dogs have increased serum triglycerides concentration

All females of control group had normal parameters in the physical and complementary examinations. The mean age values of the control and obese groups were 4.73 ± 0.52 and 5.24 ± 0.36 years old, respectively, and there was no statistical difference between the groups (p = 0.42). The obese group had a higher median for the (BCS): 9 (8; 9) × 5 (5; 5) and for the body weight: 12.4 kg (5.1 kg; 46.8 kg) × 7.5 kg (4.6 kg; 28.6 kg). The characteristics of the groups (age, weight, BCS and breed) are shown in Table 1. All other relevant characteristics from females of both groups can be found in Supplementary Table 1.

**Table 1:** Characteristics of groups.

Characteristics	Control Group (n = 11) *number of animals (%)*	Obese Group (n = 21) *number of animals (%)*
**Age (years)**		
3–5	7 (63.6%)	12 (57.1%)
6–8	4 (36.4%)	9 (42.9%)
**Weight (kg)**		
0–6	4 (36.4%)	3 (14.3%)
7–10	6 (54.5%)	7 (33.3%)
11–20	0	5 (23.8%)
21–30	1 (9.1%)	1 (4.8%)
> 30	0	5 (23.8%)
**BCS**		
5	11 (100%)	0
8	0	9 (42.7%)
9	0	12 (57.3%)
**Breed**		
Maltese	1 (9.1%)	2 (9.5%)
Dachshund	2 (18.2%)	1 (4.8%)
Mixed breed	4 (36.4%)	6 (28.6%)
Shih-Tzu	2 (18.2%)	4 (19.0%)
Rottweiler	0	1 (4.8%)
English Pointer	0	1 (4.8%)
Golden retriever	0	3 (14.3%)
Beagle	0	1 (4.8%)
Dalmatian	0	1 (4.8%)
Cocker spaniel	0	1 (4.8%)
Lhasa Apso	1 (9.1%)	0
Pinscher	1 (9.1%)	0

*n* number of animals per group.

In the urinalysis examination, there was no difference between the groups in relation to the parameters evaluated, and the females did not present glycosuria and signs of inflammation and/or infection (data not shown). The groups did not differ in terms of the blood count parameters (Table 2). In relation to the biochemical profile, obese group had a higher serum concentration of triglycerides than the control group (Table 3).

**Table 2:** Mean values of hematological findings.

Parameters	Control (n = 11)	Obese (n = 21)	p-value
Erythrocytes (10^6^/µL)	7.8 ± 0.10	7.4 ± 0.16	0.0581
Hemoglobin (g/dL)	19.2 ± 0.32	18.2 ± 0.38	0.1206
Hematocrit (%)	53.7 ± 0.94	51.5 ± 1.0	0.1767
Plasma protein (g/dL)	7.2 ± 0.29	7.3 ± 0.11	0.6972
Platelets (/µL × 10^4^)	26.9 ± 3.7	32.3 ± 2.9	0.2698
Leucocytes (/µL × 10^3^)	9.5 ± 0.81	9.7 ± 0.57	0.8395
Neutrophils (/µL × 10^3^)	5.9 ± 0.59	6.3 ± 0.39	0.3886
Eosinophils (/µL × 10^2^)	7.5 ± 1.3	7.9 ± 1.7	0.4241
Lymphocytes (/µL × 10^3^)	2.4 ± 0.49	2.2 ± 0.19	0.6082
Monocytes (/µL × 10^2^)	4.6 ± 0.83	4.3 ± 0.56	0.7298

±  standard error of mean.

The comparison between the mean values was performed using the unpaired t-test. p < 0.05.

**Table 3:** Mean values of the biochemical profile.

Parameters	Control (n = 11)	Obese (n = 21)	p value
Cholesterol (mg/dL)	210.6 ± 20.7	228.3 ± 14.2	0.4788
Triglycerides (mg/dL)	**87.4 ± 23.2**	**148.5 ± 22.7**	**0.0246**
Glucose (mg/dL)	104.8 ± 3.5	111.8 ± 2.8	0.1382
ALT (Ul/L)	46.4 ± 5.9	61.9 ± 9.4	0.3554
ALP (Ul/L)	86.8 ± 32.2	91.1 ± 33.3	0.6043
GGT (Ul/L)	4.5 ± 0.62	3.8 ± 0.63	0.1019
Total Protein (g/dL)	6.7 ± 0.28	6.6 ± 0.09	0.6873
Albumin (g/dL)	3.5 ± 0.07	3.5 ± 0.06	0.8283
Urea (mg/dL)	32.4 ± 2.4	35.9 ± 2.3	0.3361
Creatinine (mg/dL)	1.0 ± 0.06	1.0 ± 0.03	0.6654

*ALT* alanine aminotransferase, *ALP* alkaline phosphatase, *GGT* gamma glutamyl transferase,  ± standard error of mean. The comparison between the mean values was performed using the unpaired t-test. p < 0.05.

No obese female dog showed clinical signs or changes on physical examination suggestive of hyperadrenocorticism (including abnormal ultrasound evaluation of the adrenal glands) or hypothyroidism. In addition, no obese female had a low serum concentration of free T4 (Supplementary Table 2).

**Figure 1:**
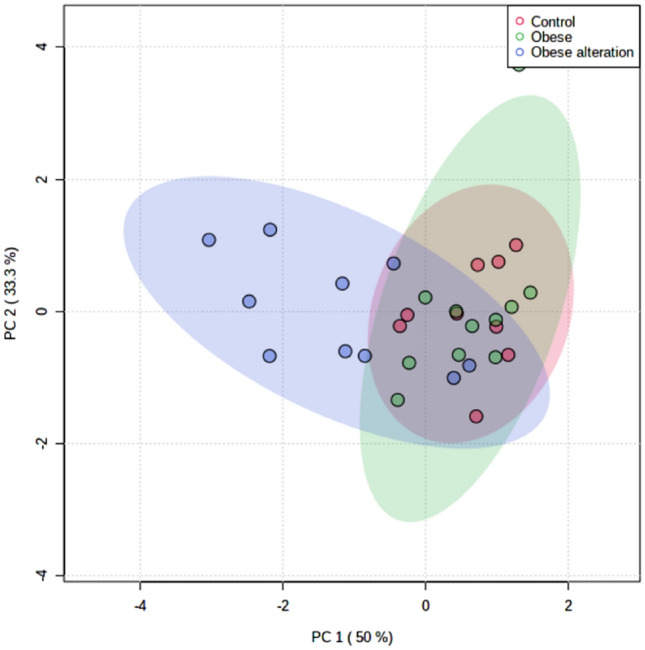
Principal component analysis (PCA) graph. The females of the obese with alteration group were assembled based on higher concentrations of small EVs and serum triglycerides.

Principal component analysis (PCA) showed that some obese female dogs were grouped together because they had higher serum triglycerides and serum sEVs concentrations (Fig. 1). Based on this analysis, these female dogs were regrouped into a third group called obese with alteration, and from that we compared the three groups: control (n = 11), obese (n = 11), and obese with alteration (n = 10).

The comparison between the three groups showed that the obese with alteration group had a higher serum concentration of triglycerides than control and obese groups (Table 4), and 70% of the female dogs had a BCS of 9.

**Table 4:** Mean values of serum concentration of cholesterol and triglycerides.

Parameters	Control (n = 9)	Obese (n = 11)	Obese with alteration (n = 10)	p value
Cholesterol (mg/dL)	186.6 ± 14.5 A	199.2 ± 12.7 A	260.6 ± 22.9 A	0.0584
Triglycerides (mg/dL)	65.1 ± 11.8 A	83.6 ± 10.5 A	**219.8 ± 34.4 B**	0.0004

± standard error of mean. The comparison between the mean values was performed using the Tukey test. Letter A on the same line indicates no statistical difference between the groups. Letter B on the same line indicates that the Obese with alteration differed from the other groups.

### Protocol efficiency for sEVs analysis

**Figure 2:**
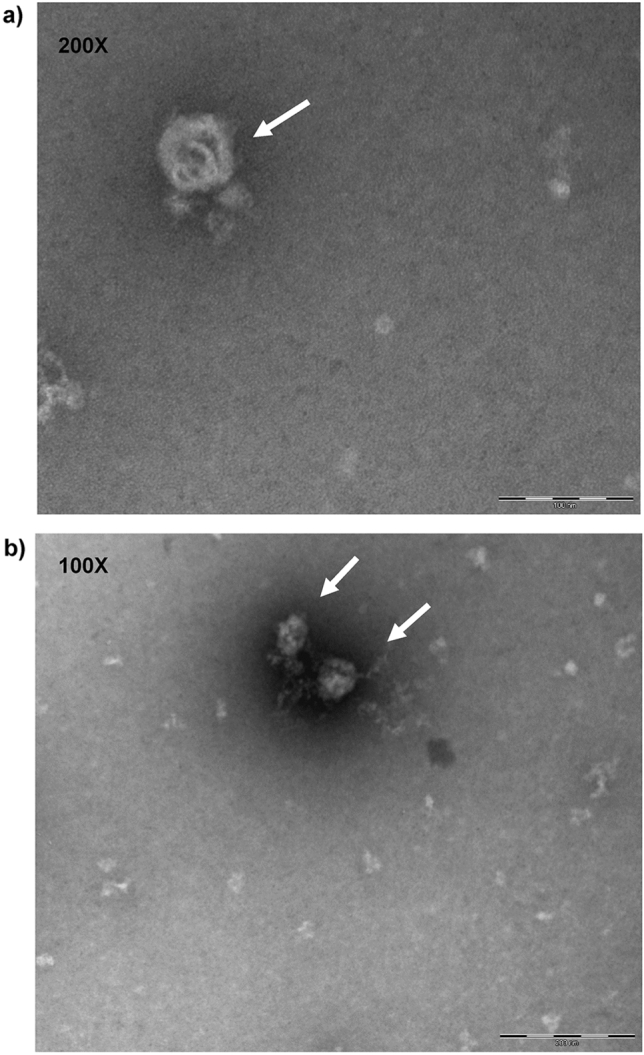
Characterization of small EVs by transmission electron microscopy. (**a**) Arrow indicates small EVs at 100 nm size. (**b**) Arrows indicate small EVs at 200 nm size.

**Figure 3:**
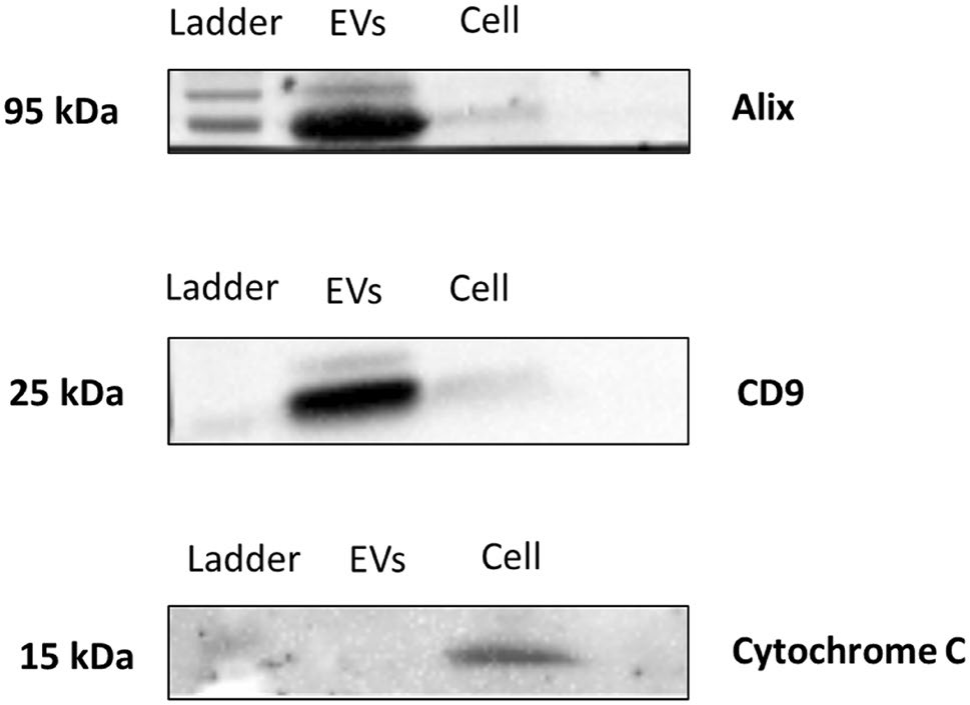
Characterization of small EVs by Western blotting. The isolated serum pellet from a female dog expressed the Alix and CD9 proteins, but it did not express Cytochrome C protein. The cell sample (canine fibroblasts cells) expressed the Cytochrome C protein. Ladder: protein molecular weight. Western blotting data are results from figures that have been sectioned without exposure manipulation, full image can be found in the Supplementary Figure S2.

The efficiency of the sEVs enrichment protocol was confirmed by transmission electron microscopy (TEM) and Western blotting. The nanoparticles identified by TEM showed shapes and sizes similar to sEVs (Fig. 2a,b, Supplementary Fig. S1). sEVs expressing the surface marker protein Alix and the inner protein CD9 and not expressing the cellular marker protein Cytochrome C (Fig. 3, Supplementary Fig. S2).

### Obese female dogs have a higher concentration of circulating sEVs and higher expression of miR-132 and miR-26b

**Figure 4:**
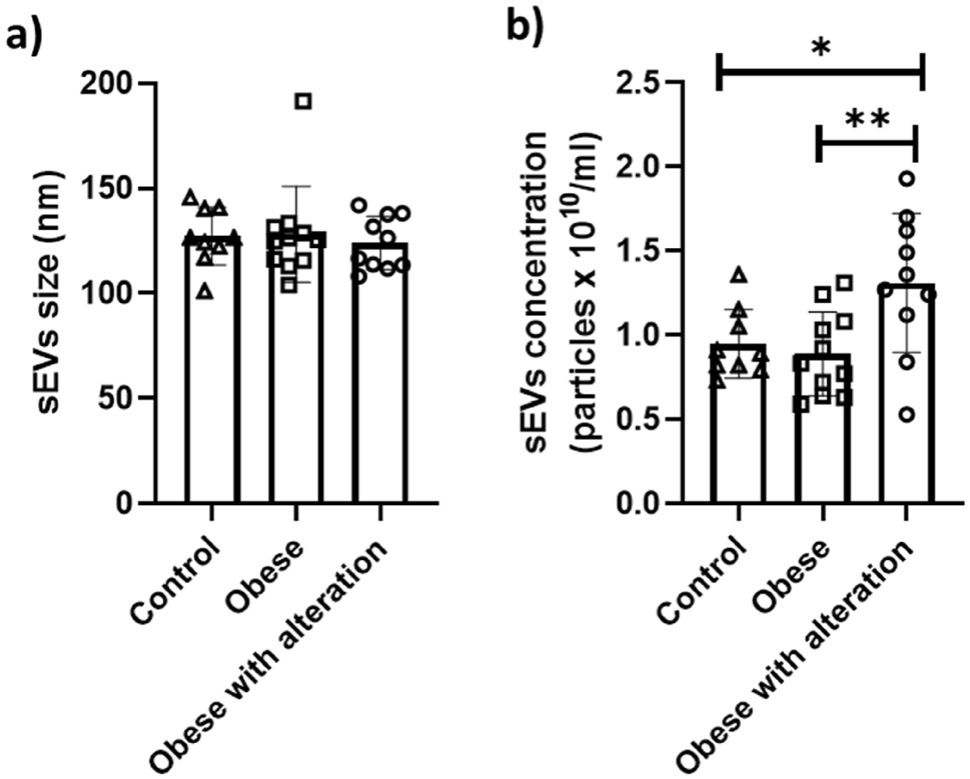
Analysis of sEVs. The graphs illustrate individual and mean values (± standard error of mean) of (**a**) sEVs size and (**b**) sEVs concentration. The comparison between the mean values was performed using the Tukey test (p < 0.01 by **comparation between Obese × Obese with alteration groups and p < 0.05 by *comparation between Control × Obese with alteration groups).

The groups did not differ in relation to the size of sEVs; however, the obese with alteration group had a higher concentration of sEVs than the other groups. The results are shown in Fig. 4.

**Figure 5:**
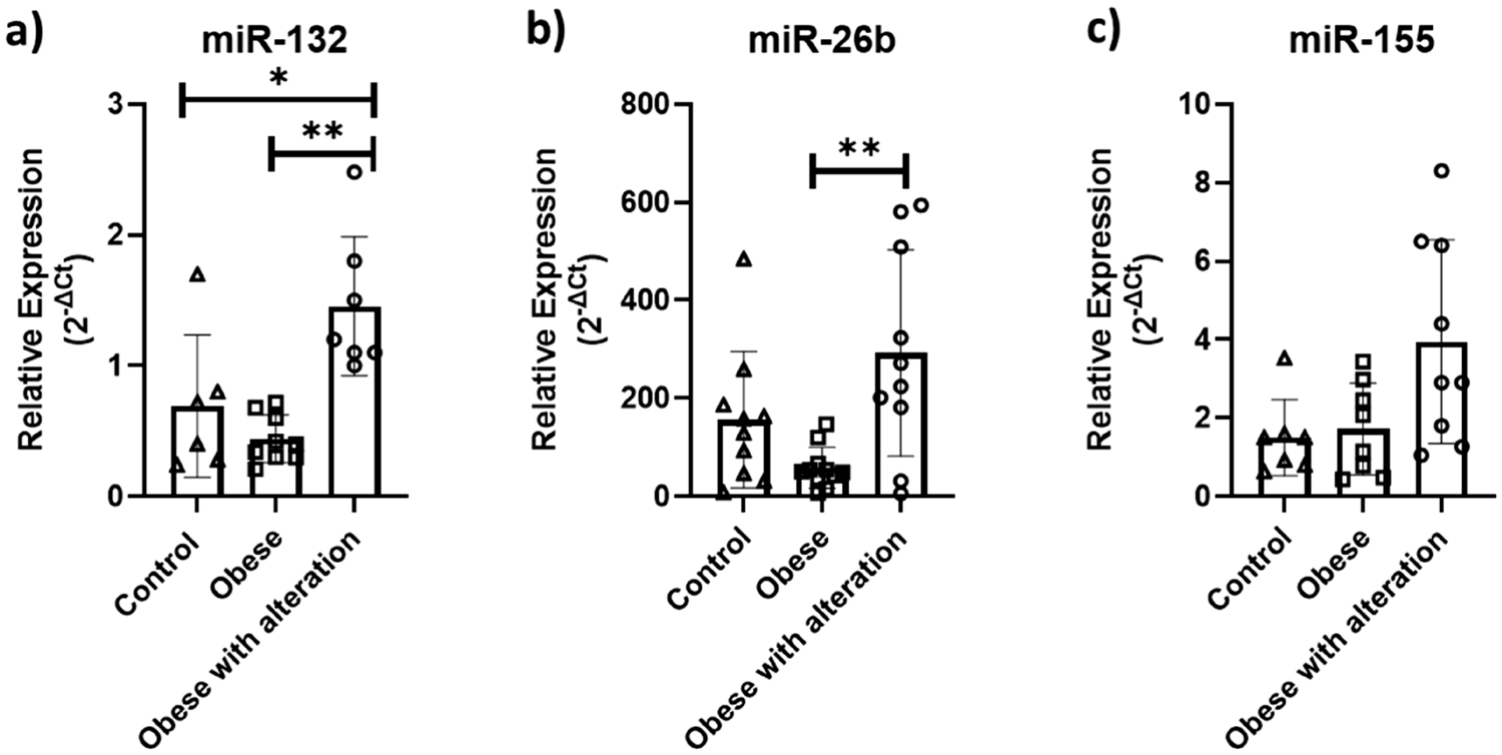
Relative expression of miRNAs. The graphs illustrate individual and mean values (± standard error of mean) of (**a**) miR-132, (**b**) miR-26b and (**c**) miR-155. The comparison between the mean values was performed using the Tukey test (p < 0.01 by **comparation between Obese × Obese with alteration groups and p < 0.05 by *comparation between Control × Obese with alteration groups).

In sEVs enriched fraction, the obese with alteration group showed higher expression of miR-132 than the other groups and higher expression of miR-26b than the obese group. However, no difference in miR-155 expression was observed between groups (Fig. 5).

## Discussion

Our study showed that some obese female dogs (10/21; 47.6%) had an increase in the serum concentration of triglycerides, which can be explained by excessive intake of calories. In addition to obesity and hypertriglyceridemia, these same females showed increased concentration of sEVs and higher expression of miRNA-132 and miRNA-26b in the sEVs enriched fraction.

Triglycerides can be derived from the diet or produced endogenously by the liver; moreover, they represent the main form of storage and transport of fatty acids within cells and in the circulation^20, 21^. Hypertriglyceridemia resulting from obesity is due: (1) to increased flow of fatty acids to the liver, with a consequent increase in the synthesis of triglyceride-rich lipoproteins, such as very low-density lipoproteins (VLDL), and (2) to reduced lipolysis of circulating triglycerides present in chylomicrons^22^.

Obese dogs due to excessive food intake, including high-fat diets, may also experience an increase in serum cholesterol concentration and the alteration involving an increase in serum concentrations of triglycerides, cholesterol, or both, is called hyperlipidemia^23, 24^. In the obese with alteration group, hyperlipidemia was characterized by hypertriglyceridemia and a tendency to increase serum cholesterol (p = 0.0584). Several studies have shown that obese dogs commonly present changes in the lipidogram, mainly characterized by an increase in the serum concentration of triglycerides^24, 25, 27–29^. Furthermore, Diez et al*.*^30^ and Jeusette et al*.*^31^ demonstrated that obese dogs subjected to weight loss showed a significant reduction in the serum concentrations of triglycerides and cholesterol. Usui et al.^26^ evaluated the profile of triglycerides and cholesterol-carrying lipoproteins in obese and overweight dogs and observed that these dogs showed an increase in cholesterol-VLDL, triglycerides-VLDL, and triglycerides-HDL (high-density lipoproteins) fractions than dogs with normal BCS.

Endocrinopathies such as hypothyroidism and hyperadrenocorticism can cause weight gain and hypertriglyceridemia in dogs^32, 33^; however, the obese female dogs recruited for this study did not present low levels of free T4 hormone nor signs of hyperadrenocorticism in the clinical evaluation.

Obese female dogs with hypertriglyceridemia (from obese with alteration group) showed an increase in the sEVs concentration. We raise the possibility that both increases in serum triglycerides and in the sEVs concentration could be related to obesity in these female dogs. This hypothesis is based on the following information: (1) adipocytes release EVs; (2) an increase in circulating levels of EVs could occur during adipose tissue expansion due to obesity and (3) obese humans with high levels of triglycerides also show an increase in circulating EVs; moreover, triglyceride levels were positively correlated with the number of circulating EVs in these individuals^13, 14, 34, 35^.

The factors that affect the pattern of circulating EVs in healthy or obese dogs are unknown. What we can assume is that the size and concentration of serum EVs did not differ between control (BCS 5) and obese (BSC 8 or 9) female dogs, suggesting that body fat accumulation alone was not sufficient to alter serum sEVs. However, the accumulation of body fat associated with hypertriglyceridemia, a consequence of obesity, resulted in a higher concentration of serum EVs.

Obesity can cause inflammation of adipose tissue, with infiltration of this tissue by immune cells and production of proinflammatory cytokines by adipocytes, which act locally or systemically^36^. Scientific evidence indicates that inflammation of adipose tissue could be a stimulus for the release of EVs by adipocytes. In this sense, in vitro studies have shown that adipocytes exposed to tumor necrosis factor alpha have increased EVs secretion^13^. In our study, it cannot be said that females from obese with alteration group had an inflammatory condition associated with obesity, which could cause a higher sEVs concentration. In part because the concentrations of serum C-reactive protein and plasma fibrinogen were evaluated in the female dogs and no difference was observed between the three groups (Supplementary Table 3).

As in this research, other studies have already evaluated EVs in canine serum samples^7, 37^. However, platelet activation occurs during coagulation, as does the spontaneous formation of platelet membrane microvesicles, which could cause an increase in EVs in the sample^4, 38^. Thus, serum does not contain only the circulating EV population. But it is important to emphasize that the comparison of the size and concentration of sEVs between the experimental groups was possible due to the standardization of blood processing and the protocol of enrichment of sEVs, and possibly obesity plus hypertriglyceridemia were the main factors that caused the observed changes.

Analysis of the enriched sEVs fraction containing miRNAs showed that obese with alteration group had higher expression of miR-132 than the other groups and miR-26b than the obese group. miRNAs, present in tissues and in circulation, are a class of non-coding RNAs that regulate gene expression by repressing translation and/or initiating the degradation of target mRNA, thus controlling several biological processes^8, 9^. Lipid metabolism is complex and can be influenced by miRNAs, mainly regarding the modulation of the expression of genes involved in cholesterol transport across cell membranes and fatty acid synthesis^39^.

miR-132 and miR-26b have been identified as miRNAs that are active in the regulation and development of adipocytes. Obesity has a great influence on adipogenesis, with pre-existing adipocytes expanding to accommodate excess nutrients in the form of triglycerides^40, 41^. A study in obese humans found that miR-132 expression in visceral adipose tissue was associated with body mass index and that miR-132 expression in subcutaneous adipose tissue was associated with plasma triglyceride levels; thus, this miRNA plays a role in the metabolic implications caused by obesity^15^. Transgenic mice overexpressing miR-132 in peripheral tissues showed increased body weight, hyperlipidemia, hepatic steatosis, and elevated liver triglycerides^41^. In the same study, antisense oligonucleotides were injected to suppress miR-132 expression in these mice, resulting in reduced hepatic steatosis and hyperlipidemia.

Studies performed in humans, mice, and cell culture have shown that miR-26b affects adipogenesis, resulting in adipocyte differentiation and triglyceride accumulation^42–44^. Song et al*.*^44^ demonstrated that miR-26b in humans is associated with adipokines and is upregulated during the differentiation of preadipocytes into adipocytes. An in vitro study evaluated the role of miR-26b in obesity, comparing preadipocytes overexpressing miR-26b with negative control cells, and observed that overexpression of miR-26b promoted adipogenesis and triglyceride accumulation in human adipocytes^43^.

The set of these scientific evidence supports the supposition that obese individuals could have alterations in lipid metabolism associated with a differentiated expression of miRNA-132 and miRNA-26b. Therefore, it is possible that the increase in the expression of miR-132 and miR-26 in the sEVs enriched fraction is somehow related to obesity and hypertriglyceridemia in female dogs. Furthermore, females with normal hematological and biochemical parameters showed no changes in sEVs and obese females showed lower expression of miR-132 and miR-26b in the sEVs enriched fraction than obese females with hyperlipidemia. These results agree with other studies that demonstrated that changes in EVs and associated miRNA were related to metabolic dysfunctions, such as hypertriglyceridemia, in obese humans^34, 35, 45^.

miR-26b, miR-132 and miR-155 can also modulate the inflammatory response^46–48^. But as previously mentioned, it is not possible to say that obesity and hypertriglyceridemia would be associated with chronic inflammation in the evaluated female dogs, even with the increased expression of miR-132 and miR-26 in the EVs enriched fraction.

EVs are produced in different cells and secreted into the extracellular environment, making it possible for them to communicate with different organs. They contain elements of their cell of origin such as lipids, proteins, and nucleic acids (mRNAs, total RNAs, and miRNAs). Thus, they act as vectors of biological information because they carry out intercellular communication and can modify the function of receptor organs^6^. In our study, we found that obese female dogs with hypertriglyceridemia had higher sEVs concentration and higher expression of miRNA-132 and miRNA-26b in the sEVs enriched fraction. However, we cannot say that adipose tissue is the origin of the sEVs analyzed in obese and control dogs, as we did not use a specific marker to identify the tissue responsible for releasing EVs. In that regard, perilipin A, which is a protein highly expressed in adipocytes, could be used as a marker of EVs released by adipose tissue^35^.

Although EVs carried different miRNAs, extracellular miRNAs also circulate in the bloodstream associated with protein complexes or lipoproteins^49^. Therefore, we cannot confirm that the miRNAs evaluated were derived only from EVs and it remains to be seen whether (1) there was a greater release of sEVs by the adipose tissue of these obese female dogs and (2) miRNAs were more expressed in the content of sEVs.

The present study had some limitations. The sEVs were obtained from canine serum samples, and even with careful blood collection, avoiding sample agitation, clotting can increase the number of EVs and interfere with the result of the sEVs concentration. We consider that the changes observed in the female dogs were caused by obesity; however, it was not possible to confirm the cellular origin of the EVs analyzed in serum samples of obese and control dogs, as specific markers were not used to identify the tissue responsible for the sEVs release. Nor can we state that miRNAs were derived exclusively from EVs.

In conclusion, obese female dogs with hypertriglyceridemia showed an increase in the serum concentration of sEVs and in the expression of miR-132 and miR-26b. No changes were observed in sEVs in female dogs with normal serum biochemical profile and in relation to miR-155 expression for all groups. These results suggest that obesity can lead to the development of hypertriglyceridemia in female dogs resulting in changes in the serum sEVs. Our study also suggests that miR-132 and miR-26b could be related to alterations in the lipid metabolism of obese female dogs.

## Methods

### Animal model

Thirty-two healthy neutered female dogs of different breeds, aged three to eight years old, were included in the experiment. Only female dogs with obesity related to excessive food intake (positive energy balance) and in this condition for at least 1 year were included in the evaluations. Healthy female dogs were used as controls. The study was conducted at the Hospital Clinical Didactic Unit at the Faculty of Animal Science and Food Engineering at the University of São Paulo. The experimental protocols performed in this study were approved and all procedures involving animals were conducted in accordance with the Ethic Committee on Animal Use of the Faculty of Animal Science and Food Engineer—FZEA—University of São Paulo, Pirassununga, SP, Brazil, composed of community members and experts in animal care and ethics (animal ethics approval number: 1940130519). All methods were reported in accordance with the ARRIVE guidelines for the reporting of animal experiments. For more details: Supplementary Material.

### Experimental groups, sample collection and complementary examinations

Female dogs with normal BCS were included in control group (n = 11). Female dogs with BCS of 8 and 9 were included in obese group (n = 21). The BCS was evaluated according to the method proposed by Laflamme^50^. After principal component analysis (PCA), the obese female dogs were regrouped into an obese group (n = 11) and an obese group with alteration (n = 10).

The females were subjected to blood collection (jugular venipuncture after 12 h of fasting) for screening laboratory tests and to analyze the size and concentration of sEVs. Blood counts were performed using an automatic blood analyzer and serum biochemical parameters were assessed using specific reagents and a biochemical analyzer. T4-free hormone dialysis test was performed at the PROVET (reference laboratory in the city of São Paulo/Brazil) with the radioimmunoassay methodology. Urine was collected by cystocentesis, and urinalysis comprised a dipstick analysis and sediment examination. Ultrasonographic evaluation was performed to rule out comorbidities, such as organ neoplasms, in addition to evaluating the adrenal glands. For more details: Supplementary Material.

### Enrichment of sEVs

Upon collection, blood samples (5 mL) were kept at room temperature for clotting and then at 4 °C for another 2 h without agitation. Subsequently, blood samples were centrifuged (2400 rpm for 30 min) to obtain the serum. Briefly, 1.5 mL of serum was centrifuged three times at 4 °C: 300×*g* for 10 min, 2000×*g* for 10 min, and 16,500×*g* for 30 min to remove cells, cell debris, and large vesicles, respectively. The supernatant was stored at − 80 °C for further ultracentrifugation. Samples were thawed and supernatants were filtered using a 0.20 μm pore polyethersulfone (PSE) membrane syringe filter to remove any large EVs. For enrichment of sEVs, the sample was subjected to two consecutive ultracentrifugation at 119,700×*g* for 70 min at 4 °C, according to a previously described protocol^51^.

The resulting pellet was resuspended in 50 µL of phosphate buffered saline (PBS) (1× Ca^2+^/Mg^2+^ free PBS). Nanoparticle tracking analysis to characterize sEVs according to particle size and concentration was performed using a NanoSight NS300 instrument (Malvern Panalytical, UK). For reading, three consecutive 30-s videos were taken for each diluted sample (1:100 in PBS) and captured by the sCMOS camera at camera level 13 at a controlled temperature of 38.5 °C. Considering threshold level 5, the images were tracked using the analytical software NanoSight NTA 3.4, as described by de Ávila et al.^52^.

### Characterization of sEVs

The presence of sEVs in canine serum was confirmed by the expression of specific proteins by Western blotting, according to a previously established protocol^37,52^ and by TEM. The primary antibodies used for Western blotting were anti-Alix produced in goat, polyclonal with reactivity in domestic dog, bovine, horse, human mouse, rat and pig (1:750; sc-49267; Santa Cruz, USA), anti-CD9 produced in mouse, monoclonal with reactivity in human, mouse and rat (1:500; sc-13118; Santa Cruz, USA), and anti-cytochrome C produced in goat, polyclonal with reactivity in domestic dog, bovine, horse, human mouse, rat and pig (1:1000; sc-8385; Santa Cruz, USA)^37^. The secondary antibody was anti-goat IgG (1:4000; sc-2020; Santa Cruz, USA). The images were obtained with ChemiDoc MP Image System and Image Lab 6.0.1 software (Bio-Rad, USA).

For TEM, sEVs isolated from 200 μL of canine serum were diluted in 200 μL of fixative solution (1% cacodylate, 2% glutaraldehyde, and 2% paraformaldehyde at pH 7.2–7.4) and maintained for 2 h at room temperature. Subsequently, the sEVs were diluted in 2 mL of 1× Ca^2+^/Mg^2+^-free PBS, and the solution was centrifuged once to obtain the sEVs pellet (119,700×*g*, 70 min, 4 °C). The pellet was diluted in 100 μL buffer solution (1% cacodylate) and refrigerated until analysis. The sEV solution was placed on a copper grid for 20 min at room temperature, and 2% uranyl acetate was added. The morphology of the sEVs was analyzed using TEM (FEI Tecnai 20; LAB6 emission; 200 kV) (Thermo Fisher Scientific, EUA).

### Total RNA extraction, reverse transcription, and real-time PCR

The total RNA content of the sEVs, including miRNAs, was extracted with the TRIzol reagent and, with the addition of 1.33 μL of the GlycoBlue coprecipitator to the aqueous phase before RNA precipitation, as previously described by da Silveira et al*.*^53^ with minimal modifications. The samples were then analyzed by spectrophotometry to verify the quantity and quality of the extracted RNA. RNA samples were treated with DNase I.

To analyze the expression of miRNAs in sEVs enriched fraction, reverse transcription was performed for cDNA synthesis using the commercial kit MystiCq MicroRNA cDNA Synthesis Mix, according to the manufacturer’s instructions. Using 70 ng of total RNA (per sample), quantitative RT-PCR was performed using the MystiCq Universal PCR Primer kit, according to the manufacturer’s instructions. Reactions of 6 μL were performed containing 2 × MystiCq microRNA SYBR Green qPCR ReadyMix, 10 μM MystiCq Universal PCR Primer, nuclease-free water, 0.7 ng cDNA, and 1 μL primer, designed according to the miRNA sequences (miR-26b, miR-132, and miR-155) available from the miRBase database (Table 5). The relative level of each miRNA in each sample was assessed, and the plates were mounted using an electronic multichannel pipetting system. Amplification was performed using the QuantStudio 6 Flex (Thermo Fisher Scientific, USA). The temperatures and times used were 95 °C for 2 min for activation of HotStarTaq DNA Polymerase and initiation of denaturation of the cDNA strands, followed by 45 cycles of 95 °C for 5 s for denaturation, 60 °C for 15 s for annealing of the primers, and 70 °C for 15 s for extension, when fluorescence capture occurred. miRNAs were considered present when they presented a cycle threshold (CT) lower than 35 in at least three biological replicates and an appropriate melting curve. CT values ​​were normalized using the geometric mean CT of the endogenous genes RNT43snoRNA and ssc-miR-99b. Relative expression values ​​were calculated using the ΔCt method, and normalized data were transformed using 2^−ΔCt^ for graphical representation of relative transcriptional levels^54^. For more details: Supplementary Material.

**Table 5:** Primer sequences of miRNAs analyzed by RT-PCR.

miRNAs	Primer sequences
miR-26b	TTCAAGTAATTCAGGATAGGTT
miR-155	TTAATGCTAATTGTGATAGGGG
miR-132	TAACAGTCTACAGCCATGGTCG
miR-99b	CACCCGTAGAACCGACCTTGCG
RNT43 snoRNA	CTTATTGACGGGCGGACAGAAAC

### Statistical analysis

Blood count and biochemical parameters, size and concentration of sEVs, and expression of miRNAs were subjected to descriptive statistical analysis and the results were presented as mean and standard error of the mean (parametric data) or median with minimum and maximum values (non-parametric data). Mean values for age, blood count and biochemical parameters, size and concentration of sEVs, and expression of miRNAs of the three groups (control, obese, and obese with alteration) were subjected to ANOVA, and compared using Tukey’s test. BCS and weight medians were compared using the Mann–Whitney test. Comparisons between the two groups (obese and control) were performed using an unpaired t-test (p < 0.05). PCA was performed with the obese and control groups and the variables used were serum triglycerides concentration and concentration of sEVs. All statistical analyses were performed using the GraphPad Prism 8.0.2 (GraphPad Software, USA).

## Supplementary Information

Supplementary Information.

## Data Availability

The data that supports the findings of this study are available in the manuscript and supplementary materials, and the datasets analyzed during the current study are available in the OMIX, China National Center for Bioinformation / Beijing Institute of Genomics, Chinese Academy of Sciences (https://ngdc.cncb.ac.cn/omix/release/OMIX001212: accession no. OMIX001212)^55^.
