# Supplementary material for: Evaluation of circulating extracellular vesicles and miRNA in neutered and obese female dogs
Source: Sci Rep. 2022 Sep 30;12:16439. doi: 10.1038/s41598-022-20523-x (PMC9525304; doi:10.1038/s41598-022-20523-x)
Supplement: Supplementary file 1 — Supplementary Information. [file 41598_2022_20523_MOESM1_ESM.docx]

**Evaluation of circulating extracellular vesicles and miRNA in neutered and obese female dogs**

Paola Caroline da Silva Nunes^1^, Rosane Mazzarella^1^, Juliano Coelho da Silveira^1^, and Deise Carla Almeida Leite Dellova^1^

^1^Faculty of Animal Science and Food Engineering, University of São Paulo, Department of Veterinary Medicine, 13635-900, Pirassununga, Brazil

Corresponding author: Deise Carla Almeida Leite Dellova

E-mail address: leite-dellova@usp.br

**Supplementary figures**

**Supplementary figure S1.** Original images of small extracellular vesicles using transmission electron microscopy (TEM).

**a)**

**

**

**200X**

**b)**

**

**

**100X**

**c)**

**

**

**50X**

Legend. Small extracellular vesicles (sEVs) visualized at 100 nm size **(a)**. sEVs visualized at 200 nm size **(b)**. sEVs visualized at 500 nm size **(c)**.

**Supplementary figure S2.** Western blotting membranes used for anti-Alix **(a),** anti-CD9 **(b)** and anti-Cytochrome C **(c)**.

**a)** anti-Alix

**
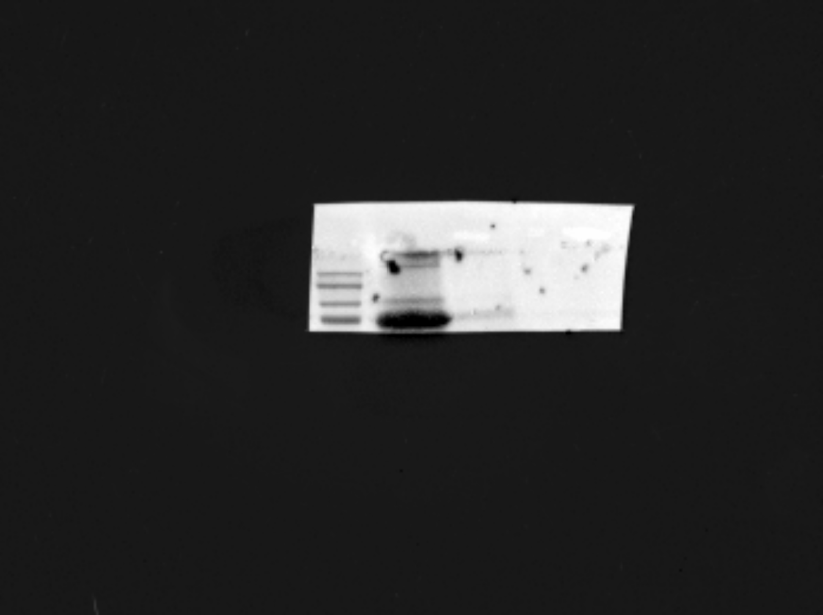
**

Alix 95kDa

Ladder EVs Cell

**b)** anti-CD9

**
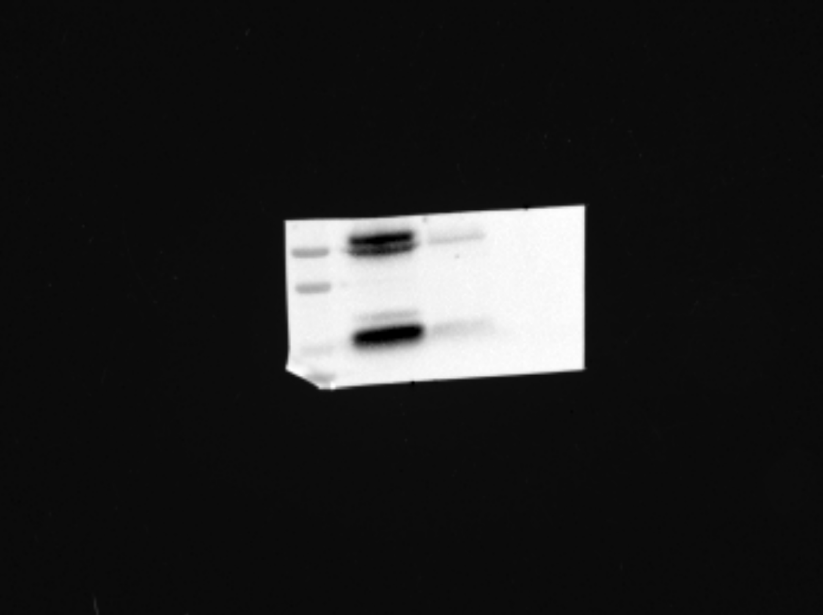
**

CD9 25kDa

Ladder EVs Cell

**c)** anti-Cytochrome C

**
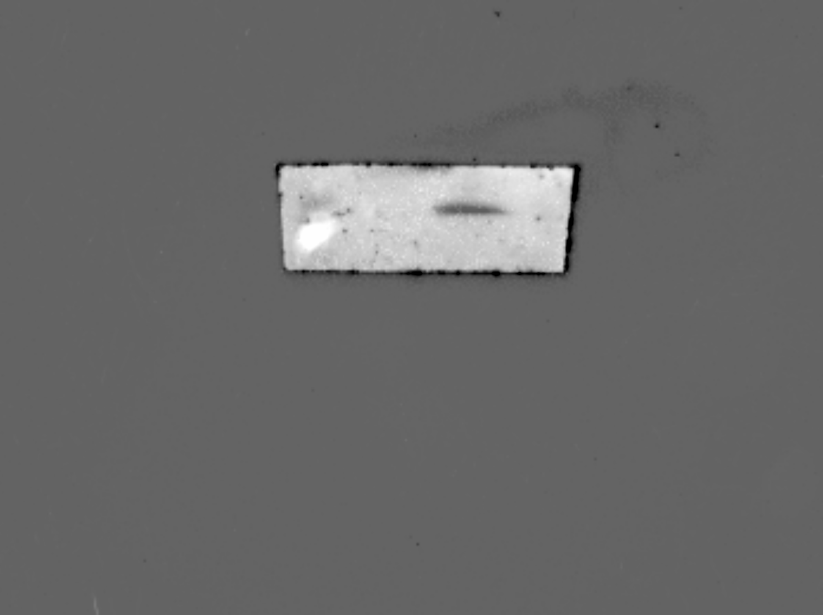
**

Ladder EVs Cell

Cytochrome C

15kDa

Legend. The isolated serum pellet from a female dog expressed the Alix **(a)** and CD9 **(b)** proteins, but it did not express Cytochrome C **(c)** protein. The cell sample (canine fibroblasts cells) expressed the Cytochrome C **(c)** protein. Ladder: protein molecular weight. The blots were cut before the addition of antibodies to optimize the gel run during the electrophoresis and the use of consumables and reagents.

**Supplementary methods**

**Animal model**

Diet: all females recruited were fed commercial food (premium and super premium) plus snacks, without control of energy intake (ad libitum).

All information regarding the animals used in the control and obese groups are presented in the Supplementary table 1.

**Supplementary table 1.** Group characteristics

| **Control Group (n=11)** | | | | |
| --- | --- | --- | --- | --- |
| **Animal Name** | **Age (years)** | **Breed** | **Weight (Kg)** | **BCS** |
| Lina | 4 | Maltese | 5.2 | 5 |
| Morena | 3 | Lhasa Apso | 8.5 | 5 |
| Nutella | 4 | Dachshund | 7.6 | 5 |
| Luna | 3 | Mixed breed | 9.4 | 5 |
| Meg | 7 | Shih-tzu | 7 | 5 |
| Bela | 6 | Mixed breed | 7.5 | 5 |
| MiniGun | 3 | Mixed breed | 28.6 | 5 |
| Amora | 4 | Dachshund | 8.2 | 5 |
| Loren | 8 | Shih-tzu | 5.9 | 5 |
| Jhully | 6 | Pinscher | 5.2 | 5 |
| Chocolate | 4 | Mixed breed | 4.6 | 5 |
| Mean | 4.73 ± 0.52 | --------- | --------- | ---- |
| Median | -------- | --------- | 7.5 (4.6; 28.6) | 5 (5; 5) |
|  |  |  |  |  |
| **Obese Group (n=21)** | | | | |
| **Animal Name** | **Age (years)** | **Breed** | **Weight (Kg)** | **BCS** |
| Kitha | 4 | Maltese | 5.1 | 8 |
| Life | 5 | Beagle | 18.2 | 9 |
| Flor | 6 | Golden retriever | 43.8 | 9 |
| Kira | 4 | Dachshund | 9.5 | 8 |
| Amy | 5 | Maltese | 6.5 | 9 |
| Frida | 8 | Shih-Tzu | 9.7 | 9 |
| Amora | 4 | Mixed breed | 14.5 | 8 |
| Hanna | 7 | Cocker spaniel | 14.6 | 9 |
| Tulipa | 6 | Mixed breed | 8.1 | 9 |
| Lizie | 5 | Shih-Tzu | 5.8 | 8 |
| Chadi | 5 | Mixed breed | 5.2 | 8 |
| Sophie | 8 | Shih-Tzu | 6.5 | 9 |
| Luna | 3 | English Pointer | 29.6 | 8 |
| Nala | 6 | Rottweiler | 46.8 | 9 |
| Bela | 4 | Shih-Tzu | 8.9 | 9 |
| Pandora | 7 | Golden retriever | 43.5 | 9 |
| Phoebe | 3 | Mixed breed | 16 | 8 |
| Mônica | 3 | Mixed breed | 9.5 | 8 |
| Bella | 3 | Golden retriever | 44 | 9 |
| Mila | 7 | Mixed breed | 12.4 | 9 |
| Maggie | 7 | Dalmatian | 32.6 | 8 |
| Mean | 5.24 ± 0.36 | ------------ | -------- | ---- |
| Median | --------- | ------------ | 12.4 (5.1; 46.8) | 9 (8; 9) |

Legend. n: number of animals per group; ±: standard error of mean.

**Experimental groups, sample collection and complementary examinations**

The Supplementary table 2 reveals the values of the free T4 test performed in obese female dogs. The Supplementary table 3 reveals the values of leukocyte, neutrophil and platelet counts, plasma concentration of fibrinogen and serum concentration of C-reactive protein in control, obese and obese with alteration groups, and the Supplementary figure S3 shows the ultrasound of the adrenal glands of these female dogs.

**Supplementary table 2.** Description of the values of the T4-free hormone dialysis test the obese group

| **Obese Group (n=21)** | |
| --- | --- |
| **Female dogs** | **T4-free (ng/dL)** |
| 1 | 1.43 |
| 2 | 3.95 |
| 3 | 1.67 |
| 4 | 1.23 |
| 5 | 2.31 |
| 6 | 2.48 |
| 7 | 3.52 |
| 8 | 1.71 |
| 9 | 2.28 |
| 10 | 1.30 |
| 11 | 1.33 |
| 12 | 1.25 |
| 13 | 2.21 |
| 14 | 2.37 |
| 15 | 0.92 |
| 16 | 1.44 |
| 17 | 1.34 |
| 18 | 1.76 |
| 19 | 1.01 |
| 20 | 1.28 |
| 21 | 2.20 |
| Reference value 0.82 – 3.65 | |

T4-free hormone dialysis test was performed at the PROVET (reference laboratory in the city of São Paulo/Brazil) with the radioimmunoassay methodology.

**Supplementary table 3.** Leukocyte, neutrophil and platelet counts, plasma concentration of fibrinogen and serum concentration of C-reactive protein in control, obese and obese with alteration groups.

| **Parameters** | **Control (n=11)** | **Obese**  **(n=11)** | **Obese with alteration (n=10)** | **p-value** |
| --- | --- | --- | --- | --- |
| Leukocytes (/µL x 10^3^) | 8.8 ± 0.8 | 9.6 ± 0.7 | 10.4 ± 0.8 | 0.3140 |
| Neutrophils (/µL x 10^3^) | 5.3 ± 0.2 | 5.8 ± 0.5 | 7.0 ± 0.5 | 0.0520 |
| Platelets (/µL x 10^4^) | 25.6 ± 3.9 | 28.0 ± 2.9 | 37.0 ± 4.8 | 0.1196 |
| Fibrinogen (mg/dL) | 284.6 ± 38.9 | 235.0 ± 36.4 | 230.8 ± 45.7 | 0.6045 |
| C-reactive protein (mg/dL) | 4.7 ± 1.5 | 5.4 ± 0.8 | 3.9 ± 0.8 | 0.6013 |

n: number of animals per group; ±: standard error of the mean. Mean values were compared using the Tukey test. Plasma fibrinogen concentration was determined by Clauss fibrinogen assay, using a commercial kit (Labtest Diagnostica, Brazil). Serum concentration of C-reactive protein was determined by enzyme immunoassay.

**Supplementary figure S3**. Representative images of ultrasound examination of the adrenal glands

**a) b)**


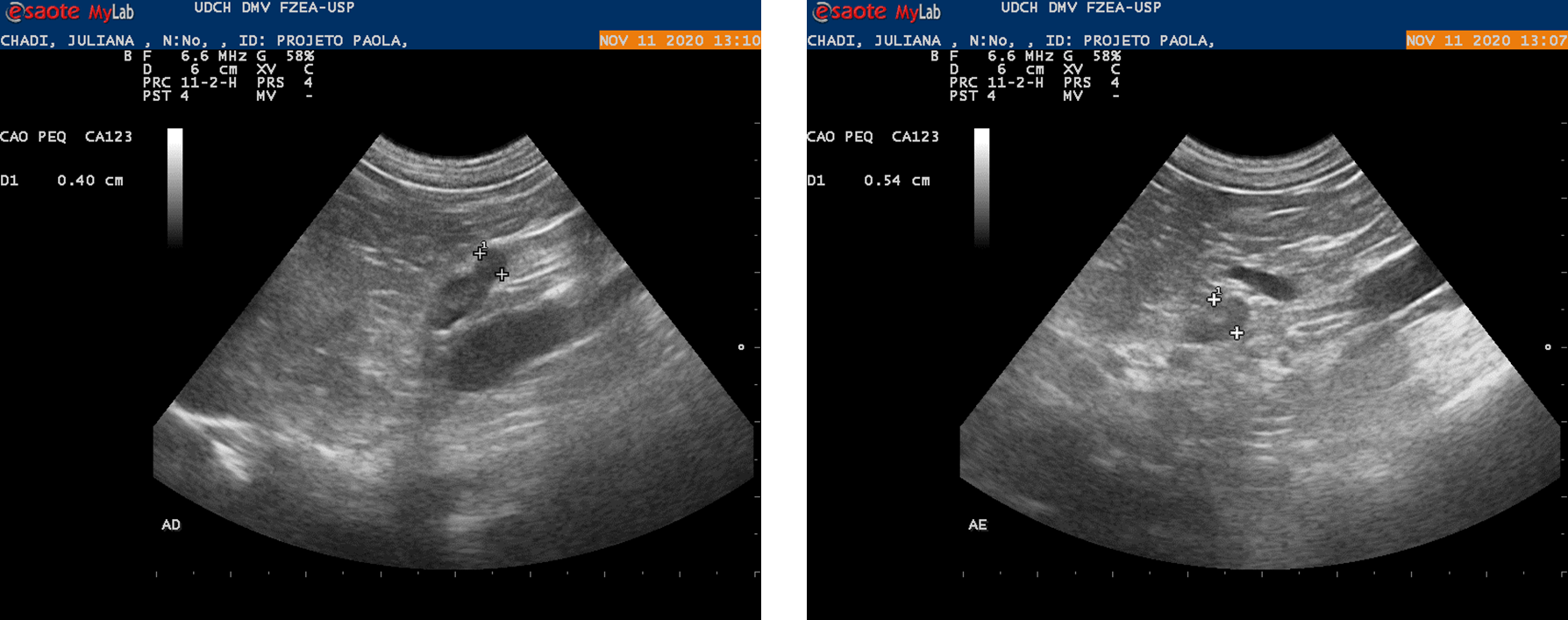


Ultrasound of the right **(a)** and left **(b)** adrenal gland. Markings (+) demonstrate diameter measurements at the caudal pole of each gland. Note that there is no deformity of the glands.

**Total RNA extraction, reverse transcription, and Real-Time PCR**

**Supplementary table 4.** Homolog between swine miRNA (ssc-miRNA) and canine miRNA (cfa-miRNA)

| **ssc-miRNA** | **Sequence** | **cfa-miRNA** | **Sequence** |
| --- | --- | --- | --- |
| **miR-26b-5p** | UUCAAGUAAUUCAGGAUAGGUU | **miR-26b** | UUCAAGUAAUUCAGGAUAGGUU |
| **miR-155-5p** | UUAAUGCUAAUUGUGAUAGGGG | **miR-155** | UUAAUGCUAAUUGUGAUAGGGGU |
| **miR-132** | UAACAGUCUACAGCCAUGGUCG | **miR-132** | UAACAGUCUACAGCCAUGGUCGC |
| **miR-99b** | CACCCGUAGAACCGACCUUGCG | **miR-99b** | CACCCGUAGAACCGACCUUGCG |
